# Deficiency of PSRC1 accelerates atherosclerosis by increasing TMAO production via manipulating gut microbiota and flavin monooxygenase 3

**DOI:** 10.1080/19490976.2022.2077602

**Published:** 2022-05-25

**Authors:** Tiantian Luo, Zhigang Guo, Dan Liu, Zhongzhou Guo, Qiao Wu, Qinxian Li, Rongzhan Lin, Peier Chen, Caiwen Ou, Minsheng Chen

**Affiliations:** a Department of Cardiology, Laboratory of Heart Center, Zhujiang Hospital, Southern Medical University, Guangzhou, China; b Guangdong Provincial Key Laboratory of Shock and Microcirculation, Guangzhou, China; c Department of Cardiology, State Key Laboratory of Organ Failure Research, Guangdong Provincial Key Lab of Shock and Microcirculation, Nanfang Hospital, Southern Medical University, Guangzhou, China; d Department of Cardiology, Huiqiao Medical Center, Nanfang Hospital, Southern Medical University, Guangzhou, China; e Department of Pharmacy, Zhujiang Hospital, Southern Medical University, Guangzhou, China; f Guangdong Provincial Key Laboratory of Shock and Microcirculation, Dongguan Hospital of Southern Medical University, Southern Medical University, Guangzhou, China

**Keywords:** Proline/serine-rich coiled-coil protein 1, trimethylamine N-oxide, gut microbiota, flavin monooxygenase 3, atherosclerosis

## Abstract

Maladaptive inflammatory and immune responses are responsible for intestinal barrier integrity and function dysregulation. Proline/serine-rich coiled-coil protein 1 (PSRC1) critically contributes to the immune system, but direct data on the gut microbiota and the microbial metabolite trimethylamine N-oxide (TMAO) are lacking. Here, we investigated the impact of PSRC1 deletion on TMAO generation and atherosclerosis. We first found that PSRC1 deletion in apoE^−/−^ mice accelerated atherosclerotic plaque formation, and then the gut microbiota and metabolites were detected using metagenomics and untargeted metabolomics. Our results showed that PSRC1 deficiency enriched trimethylamine (TMA)-producing bacteria and functional potential for TMA synthesis and accordingly enhanced plasma betaine and TMAO production. Furthermore, PSRC1 deficiency resulted in a proinflammatory colonic phenotype that was significantly associated with the dysregulated bacteria. Unexpectedly, hepatic RNA-seq indicated upregulated flavin monooxygenase 3 (FMO3) expression following PSRC1 knockout. Mechanistically, PSRC1 overexpression inhibited FMO3 expression *in vitro*, while an ERα inhibitor rescued the downregulation. Consistently, PSRC1-knockout mice exhibited higher plasma TMAO levels with a choline-supplemented diet, which was gut microbiota dependent, as evidenced by antibiotic treatment. To investigate the role of dysbiosis induced by PSRC1 deletion in atherogenesis, apoE^−/−^ mice were transplanted with the fecal microbiota from either apoE^−/−^ or PSRC1^−/−^apoE^−/−^ donor mice. Mice that received PSRC1-knockout mouse feces showed an elevation in TMAO levels, as well as plaque lipid deposition and macrophage accumulation, which were accompanied by increased plasma lipid levels and impaired hepatic cholesterol transport. Overall, we identified PSRC1 as an atherosclerosis-protective factor, at least in part, attributable to its regulation of TMAO generation via a multistep pathway. Thus, PSRC1 holds great potential for manipulating the gut microbiome and alleviating atherosclerosis.

## Introduction

Although the main therapeutic strategies for coronary artery disease (CAD), dominated by crosstalk between lipid accumulation and inflammation, are mainly based on the correction of abnormal cholesterol levels, residual risks of major adverse cardiovascular events (MACEs) remain.^1^ The recent verification of the link between the gut microbiota and lipid metabolism and inflammation has broadened our horizons that how the gut microbiota may interact with their host and diseases.

Indeed, numerous studies have demonstrated that perturbation of the gut microbiome has profound impacts on atherogenesis.^2,3^ Despite controversial descriptive data, mechanistic studies imply that specific microbial pathogens such as *Helicobacter* and *Ruminococcus* could increase lipid levels and CAD risks, while some probiotics such as *Akkermansia muciniphila* (*A. muciniphila), Bifidobacterium* and *Lactococcus* could directly or indirectly regulate the immune system and improve the gut microbiota structure.^4^ Fecal microbiota transplantation (FMT) studies have demonstrated the potential participation of dysbiosis in atherosclerosis susceptibility.^5^ As a major endocrine organ, the gut microbiota also generates toxic metabolites that accelerate atherosclerotic progression. Choline, carnitine and betaine are metabolized to produce trimethylamine (TMA) via the cut and cnt families of microbial enzymes, and then TMA travels to the liver, where it is converted into trimethylamine N-oxide (TMAO) by hepatic flavin monooxygenase 3 (FMO3). TMAO promotes atherosclerotic lesion formation by increasing foam cell formation within plaques, promoting inflammation and stimulating platelet hyperreactivity.^6,7^ Considerable evidence also indicates a positive association between elevated plasma TMAO levels and MACE risks.^8^ Hence, a better understanding of TMAO generation may reveal new strategies to prevent CAD.

Atherosclerosis is a complex process that involves the interplay between genes and the gut microbiota. However, major knowledge gaps exist because most studies have focused on the gut microbiota composition in the context of CAD rather than on the origin of these changes, or on the influences of environmental factors rather than on the host genome itself, on an individual’s gut microbiota. Proline/serine-rich coiled-coil protein 1 (PSRC1) is a microtubule-associated protein that regulates spindle dynamics, and it plays a vital role in the cell cycle and proliferation.^9,10^ The earliest genome-wide association study reported that PSRC1 single-nucleotide polymorphisms (SNPs) are strongly linked to increased susceptibility to CAD.^11^ Further studies have investigated that the locus that harbors the PSRC1 gene has a significant association with both nonfatal acute myocardial infarction risk and lipid accumulation in hepatocytes.^12,13^ In addition, PSRC1 SNPs also affect many pathological processes, including the severity of coronary stenosis, LDL-C response to statins, and variability in warfarin dosage.^14–16^ Our previous report first demonstrated a reduction in atherosclerotic lesions in apoE^−/−^ mice infected with a recombinant PSRC1-overexpression adenovirus by reducing serum cholesterol levels and inhibiting NF-κB-mediated inflammation.^17^ PSRC1 also affects circulating levels of progranulin, which is a secreted protein that regulates the immune system.^18^ Indeed, accumulating studies have revealed an important role of inflammatory and immune responses in maintaining gut barrier integrity and function.^19,20^ Lipopolysaccharide (LPS) increases murine gut permeability and gut inflammation,^21^ and the release of proinflammatory cytokines is a major contributor to gut barrier failure.^22^ Based on these facts, we proposed that the dysregulation of PSRC1 via the genetic manipulation of mice would alter the gut microbiota composition and function and accordingly affect TMAO generation.

To address this hypothesis, we first confirmed an atherosclerosis-protective role of PSRC1 in high-fat diet (HFD) fed-mice models. Then, the gut microbiome, metabolites and hepatic enzymes were examined in mice supplemented with normal- or high-dose choline diets. We found that PSRC1 deletion increased plasma TMAO levels via a metaorganismal pathway. Then, FMT was performed to clarify the underlying influence of microbial gut dysbiosis induced by PSRC1 deletion on atherosclerosis. Our studies reveal an emerging role for global PSRC1 in TMAO generation and provide potential therapeutic approaches for atherosclerosis.

## Results

### PSRC1 deletion accelerates HFD-induced atherosclerosis

**Figure 1.**
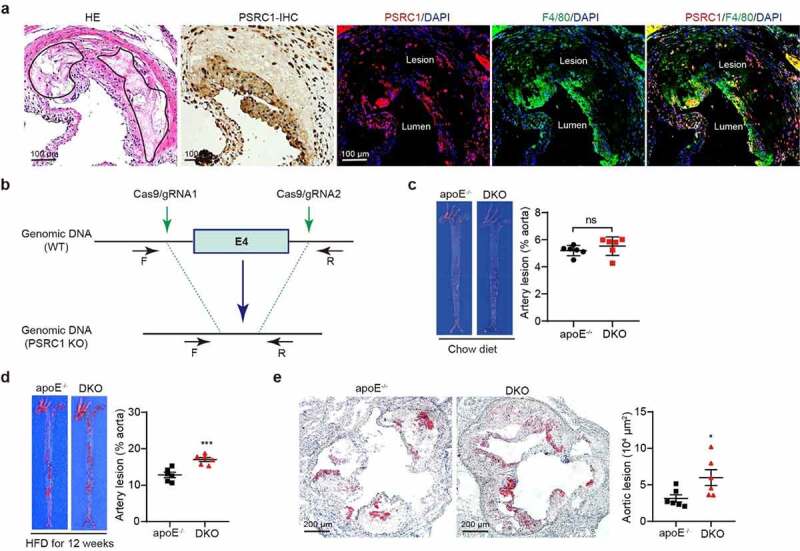
**PSRC1 deletion accelerated atherosclerotic plaque formation**. (a) Cross-sections of the mouse atherosclerotic aortic roots were stained with HE for necrotic core, immunohistochemistry and immunofluorescence for PSRC1. Representative immunofluorescence images demonstrating staining for PSRC1 in F4/80+ area of atherosclerotic aortic root sections. (b) Strategy for the generation of PSRC1^−/−^ mice. PSRC1^−/−^ mice were generated using the CRISPR–Cas9 system to delete exon 4. (c) Representative images of the *en face* aortas following oil red O staining in 8-week-old male chow diet-fed apoE^−/−^ and DKO mice (*n* = 3). (d-e) 8-week-old male apoE^−/−^ and DKO mice were treated with HFD for 12 weeks and plaque formation was assessed by oil red O staining of the *en face* aortas (d) and aortic roots (e), *n* = 6. The quantification was calculated using ImageJ software. Data are shown as the mean ± SEM. * p < .05, *** p < .001 vs. the indicated groups.

Although our previous study revealed an association between PSRC1 and atheroscleros,^17^ there is no intuitive data to reveal the distribution of PSRC1 in atherosclerotic plaques. We first described the expression of PSRC1 in murine atherosclerotic plaque samples. Strikingly, immunofluorescence staining identified PSRC1^+^F4/80^+^ macrophages within the plaque intima (Figure 1a). To further investigate the role of PSRC1 on atherogenesis, PSRC1^−/−^ mice were generated by deleting exon 4 (Figure 1b), and then were crossed with atherosclerosis-prone apoE^−/−^ mice (i.e., DKO), while apoE^−/−^ mice served as controls. The baseline results showed no distinct atherosclerotic plaques in both young chow diet-fed groups (Figure 1c). The atherosclerotic lesions were evaluated in mice receiving an atherogenic HFD for 12 weeks. Notably, we found that PSRC deletion caused a significant elevation in atherosclerotic plaque area (approximately 1.33-fold increase in the *en face* aortas and 1.91-fold in aortic root sections) (Figures 1d,1e). Thus, PSRC1 deficiency accelerates the development of HFD-induced atherosclerosis.

### PSRC1 deletion disturbs the gut microbial composition and function

**Figure 2.**
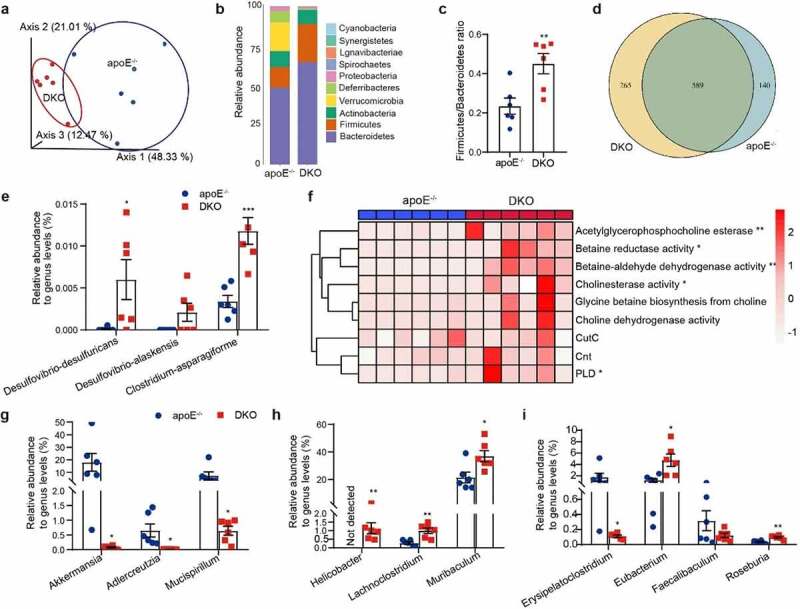
**PSRC1 deletion disturbed the gut microbiome and increased TMA-producing bacteria abundance**. The fecal samples collected from 8-week-old male chow diet-fed apoe^−/−^ and DKO mice were sequenced using metagenomics. (a) Three-dimensional pcoa showed differences in the gut microbiota composition. (b) Barplot of both groups showed the different gut microbial composition at the phylum level. (c) Ratio of firmicutes to bacteroidetes. (d) Venn plot showed the specific and common species in gut microbiota. (e) Relative abundance of tma-producing bacteria (percentage of total gut microbiota). (f) Relative abundance changes of genes involved microbial metabolic pathways converted betaine and choline to TMA summarized by gene ontology (GO) annotations, such as betaine transport, betaine reductase and choline dehydrogenase activity, and TMA synthesis-related genes summarized by KEGG orthology (ko entries) annotations, such as *cutc, cnt* and *PLD*. (g) Relative abundance of decreased bacterial genera. (h) Relative abundance of increased bacterial genera. (i) Relative abundance of SCFAs-producing bacteria. all *n* = 6 mice/group. Red dot represents DKO mice and blue represents apoE^−/−^ controls. Data are shown as the mean ± SEM. * p < .05, ** p < .01, *** p < .001 vs. apoE^−/−^ controls.

Considering the association between gut microbiota and atherosclerosis,^2,3^ we sought to investigate the impact of PSRC1 deletion on the gut microbiota, especially the TMAO generation-related bacteria. We sequenced the fecal microbiome in either male apoE^−/−^ or matched-DKO mice fed a normal chow diet (containing 0.2% total choline) using metagenomics. Principal component analysis (PCoA) revealed a largely distinct separation in gut microbiota composition (Figure 2a). At the phylum level, DKO mice exhibited less diversity, and most gut commensal bacteria in apoE^−/−^ mice belonged to the phyla Bacteroidetes, Verrucomicrobia and Firmicutes, which, along with Actinobacteria and Deferribacteres, represented approximately 95% of the total community, but only three major phyla (Bacteroidetes, Firmicutes, and Actinobacteria) were present in DKO mice (Figure 2b). PSRC1 deficiency significantly increased the ratio of Firmicutes to Bacteroidetes (0.45 versus 0.24 in apoE^−/−^ mice, p < .01) (Figure 2c), which is considered as a hallmark of obesity and atherosclerosis.^23^ Overall, the difference in the microbiota consisted of 589 common species, 140 endemic species in apoE^−/−^ mice and 256 in DKO mice (Figure 2d). The TMA-producing bacteria *Desulfovibrio-desulfuricans, Desulfovibrio-alaskensis* and *Clostridium-asparagiforme* were significantly enriched in DKO mice (Figure 2e). Functionally, DKO mouse metagenomes were enriched in genes encoding proteins involve in TMA synthesis, including phospholipase D (*PLD*), acetylglycerophosphocholine esterase, betaine reductase, betaine-aldehyde dehydrogenase and cholinesterase activity; however, no significant changes were observed for the gut microbial TMA-lyases CutC and cnt (Figure 2f).

In addition, the nearly complete depletion of the phylum Verrucomicrobia in DKO mice (0.76% versus 17.99% in apoE^−/−^ mice) was due to the significant reduction in the beneficial species *A. muciniphila*, the only representative microbe of phylum Verrucomicrobia in the mammalian gut.The genera *Adlercreutzia* and *Mucispirillum* were similarly enriched in apoE^−/−^ mice (Figure 2g). Conversely, *Helicobacter* was undetectable in apoE^−/−^ controls, whereas a bloom was observed in the DKO group, which was dominated by non-*H. pylori Helicobacter* species (Figure 2h). Gastric *H. pylori* was likewise enriched in 1 fecal sample from DKO mice (Table S1). At the functional level, we observed a significant enrichment in the acid acclimation gene *ureE*, the pH-gated urea channel-encoding gene *ureI* and genes encoding enzymes with urease activity in the gut metagenomes of DKO mice (Figure S1 and Table S2). Parallel analyses also showed that PSRC1-knockout mice displayed a higher abundance of the cholesterol-related bacterium *Lachnoclostridium* and the commensal microorganism *Muribaculum* (Figure 2h). However, short-chain fatty acid (SCFA)-producing bacteria, including *Erysipelatoclostridium, Eubacterium, Faecalibaculum* and *Roseburia*, exhibited conflicting results (Figure 2i).

To identify the enriched metabolic functions in the gut metagenome, we determined the extent of enrichment of different KEGG and GO pathways. The samples from DKO mice exhibited less potential for linoleic acid metabolism, bile acid biosynthesis and arginine metabolism and increased potential for cardiovascular diseases (Figure S2a-b). Further screening demonstrated that PSRC1 deletion reduced the abundance of ArgG due to a significant reduction in *A. muciniphila* and a non-significant reduction in *Bifidobacterium pseudolongum* (Figure S2c).

### Altered colonic inflammatory phenotypes are associated with microbial gut dysbiosis

**Figure 3.**
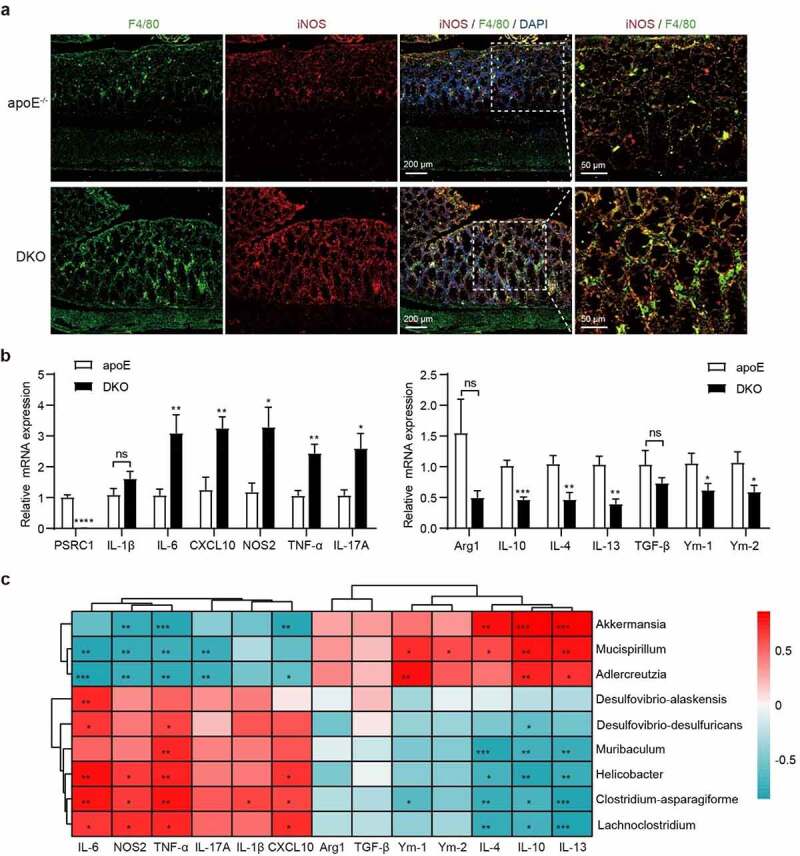
**The pro-inflammatory colonic phenotype following PSRC1 knockout was associated with the dysregulated bacteria**. (a) Immunofluorescence of colonic sections demonstrating staining for iNOS in F4/80^+^ macrophages. (b) Relative mRNA levels of pro-inflammatory markers and anti-inflammatory cytokines (to β-actin) in the colon tissues were assessed by qRT-PCR (*n* = 6). Data are shown as the mean ± SEM. * p < .05, ** p < .01, *** p < .001, **** p < .0001 vs. apoE^−/−^ mice. (c) Pearson correlations between TMA-producing bacteria and other significantly disturbed microbiota and established inflammatory markers were analyzed. The color scale is indicative of the strength of correlation, ranging from −0.5 (strong negative correlation) to 0.5 (strong positive correlation).

To investigate the role of PSRC1 in the intestinal immune and inflammatory microenvironments, we measured the colonic expression of various inflammation-related markers. Immunofluorescent staining in colonic sections revealed marked infiltration of F4/80-positive macrophages with the inflammatory phenotypes of production of inducible nitric oxide synthase (iNOS) and phosphorylated nuclear factor-kappa B (pNF-κB) p65 following PSRC1 knockout (Figure 3a and S3). These changes were accompanied by increased mRNA levels of inflammatory genes, including IL-6, IL-10, NOS2, TNF-α and IL-17A, whereas the expression of anti-inflammatory cytokines, including IL-10, IL-4, IL-13, Ym-1 and Ym-2 was significantly decreased (Figure 3b).

To further examine whether these changed levels of markers are associated with the microbial gut dysbiosis, correlation analysis was performed (Figure 3c). Fecal TMA-producing bacteria (especially *Desulfovibrio-desulfuricans* and *Clostridium-asparagiforme*) exhibited a positive correlation with proinflammatory markers (including IL-6, NOS2, TNF-α and CXCL10), but a negative correlation with anti-inflammatory markers (including IL-4, IL-10 and IL-13). The results also revealed a negative correlation between proinflammatory genes and the relative abundance of *Akkermansia, Adlercreutzia* and *Mucispirillum*.

### PSRC1 deletion upregulates hepatic FMO3 expression via the ERα-mediated pathway

**Figure 4.**
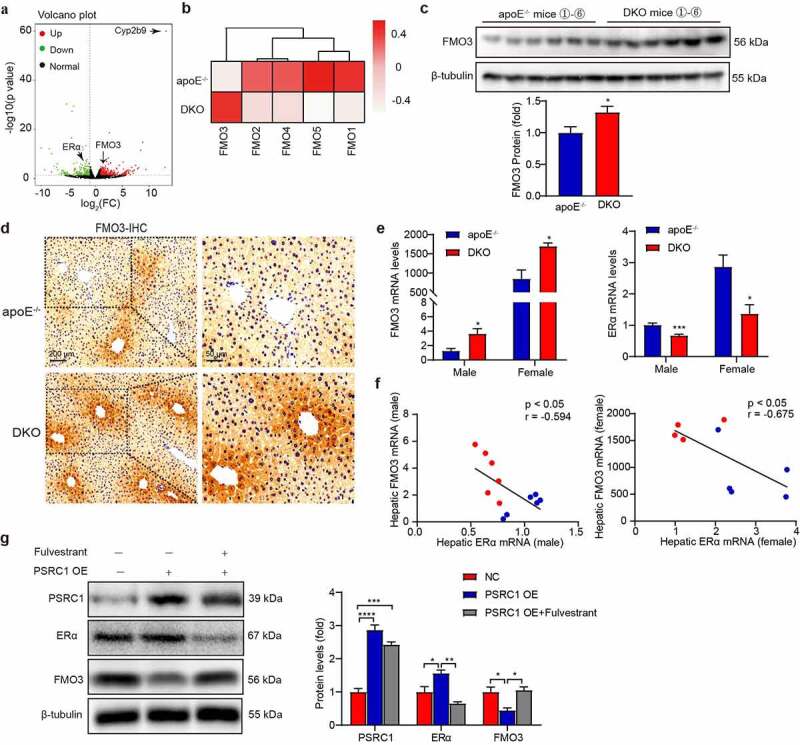
**PSRC1 deletion upregulated hepatic FMO3 expression**. Liver tissues were isolated from 8-week-old male chow diet-fed apoE^−/−^ and DKO mice for further analysis. (a) Volcano plot of RNA-seq depicting the differentially expressed genes in liver samples from DKO mice relative to those from apoE^−/−^ mice. The cutoff values for fold change and adjusted p-value were 2 and 0.05, respectively. Non-changed genes are shown in black color. Red color represents upregulated genes while green represents downregulated genes. (b) Heat map of hepatic flavin monooxygenases (FMOs) sequencing of DKO mice livers in comparison with apoe^−/−^ livers (*n* = 3). (c) Quantification of FMO3 expression was assessed by Western blot. (*n* = 6). (d) The localization of specific protein in liver was detected with antibodies against FMO3 and visualized by immunohistochemical staining. (e) Hepatic mRNA levels of FMO3 and ERα were confirmed by qRT-PCR in both male and female mice. (*n* = 4–6). (f) Correlations between FMO3 and ERα levels were analyzed. Pearson correlation coefficients (r) and p values are presented in the graph. (g) AML12 cells transfected with a negative control (NC) or targeting PSRC1-overexpressed adenovirus (PSRC1 OE) were further incubated with 5 uM Fulvestrant for 24 h. Expression of PSRC1, ERα and FMO3 were assessed by Western blot (*n* = 3). Data are shown as the mean ± SEM. * p < .05, ** p < .01, *** p < .001 vs. the indicated groups.

To clarify the role of PSRC1 deletion in hepatic synthesis and metabolic function, we performed transcriptome analysis in the livers of chow diet-fed apoE^−/−^ and DKO mice. A total of 641 genes (398 upregulated and 243 downregulated) were differentially expressed, based on a 2-fold cutoff (Figure 4a). Intriguingly, PSRC1 deletion caused a significant induction of the expression of FMO3 (Figure 4b), which is a major hepatic enzyme responsible for the conversion of TMA to TMAO. Western blot analysis confirmed this upregulation (Figure 4c). Parallel immunohistochemical studies revealed that PSRC1 deletion increased the localization of FMO3 around the central vein of hepatic lobules (Figure 4d). Similar to the previous findings that androgens downregulated FMO3 expression in mice,^24^ we observed a > 1000-fold increase of FMO3 levels in female mice relative to males (Figure 4e). Furthermore, lack of PSRC1 increased of FMO3 mRNA levels in both male and female mice (2.85- and 1.99-fold, respectively).

The bile acid receptor nuclear farnesoid X receptor (FXR) induces FMO3 expression.^24^ Therefore, we assessed the expression of FXR and the target gene bile salt export pump (BSEP), but no corresponding upregulation was found (Figure S4). Further analysis of the differentially expressed genes revealed that the transcriptional levels of estrogen receptor alpha (ERα) were significantly decreased following PSRC1 knockout (p = .027, log2FC = −0.824) (Figure 4a), which verified direct binding to the promoter region of mouse FMO3.^25^ As expected, a reduction in ERα levels after PSRC1 knockout was observed, which exhibited negative correlations with FMO3 levels in both male and female mice (Figures 4e,4f). We confirmed these results in murine hepatocytes. AML12 cells were transfected with a recombinant adenovirus encoding PSRC1 (PSRC1 OE) and the best transfection efficiency was observed at an MOI at 200 (Figure S5a). Compared with the PBS- or negative control (NC)-treated cells, the AML12 cells treated with PSRC1 OE showed a significant upregulation of PSRC1 mRNA levels (Figure S5b). In contrast, overexpression of PSRC1 strongly inhibited the levels of FMO3 (Figure 4g). Fulvestrant, a selective estrogen receptor inhibitor, has been approved for the treatment of breast cancer by inhibiting ERα signaling and suppressing the immune response.^26^ To explain the underlying mechanism by which PSRC1 OE inhibited FMO3, we used Fulvestrant to treat the transfected cells. We found that blocking ERα reversed the inhibited effect of PSRC1 OE on FMO3 (Figure 4g). These data demonstrated that PSRC1 inhibited FMO3 expression via the ERα-mediated signaling.

### PSRC1 deletion increases plasma betaine and TMAO levels

**Figure 5.**
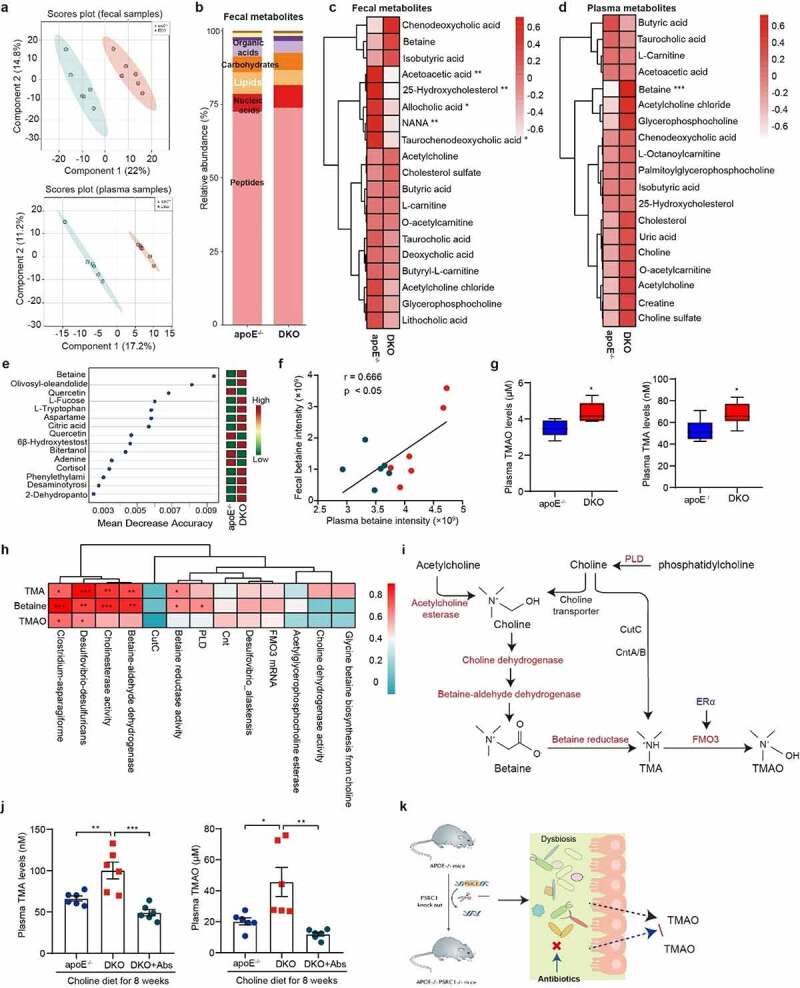
**PSRC1 deletion increased plasma levels of TMAO and betaine**. Fecal and blood samples collected from 8-week-old male chow diet-fed apoE^−/−^ and DKO mice were detected using untargeted metabolomics. (a) PLS-DA score plot showed the significant differences in metabolite composition and structure in both fecal and plasma samples. Each data point represents one sample. Red dot represents DKO mice and blue represents apoE^−/−^ controls. (b) PSRC1 deletion tended to decrease the proportion of fecal lipids (7.33% vs 5.10%). (c-d) Relative abundance of fecal (c) and plasma (d) metabolites involved choline, bile acids, SCFAs and other atherosclerosis-related substances. (e) Distribution of the discriminative markers in plasma analysts for DKO classification was shown using random forest methods. Left plot listed the most important 15 metabolites. The bigger of value of x-axis, the more important of metabolites. Right plot represents the heatmap of corresponding metabolites abundance. (f) Correlation between plasma betaine and fecal betaine intensity was analyzed. Pearson correlation coefficients (r) and p values are presented in the graph. (g) Plasma levels of TMAO and TMA were determined by LC-MS. All *n* = 6 mice/group. Data are shown as the mean ± SEM. * p < .05, ** p < .01, *** p < .001 vs. the indicated groups. (h) Correlations between the levels of TMAO, TMA and TMAO and TMA precursors and TMA-producing bacteria were analyzed. The color scale is indicative of the strength of correlation, ranging from 0 (no significant correlation) to 0.8 (strong positive correlation). (i) Pathways for TMAO and betaine generation in the present study. Red letter indicates the upregulated pathways following PSRC1 deletion. (j) 8-week-old male apoE^−/−^ and DKO mice were fed a 1% choline diet with or without antibiotic compounds (ampicillin 1 g/L, metronidazole 1 g/L, vancomycin 1 g/L and neomycin 1 g/L) for 8 weeks. Blood samples were collected and the levels of TMA and TMAO were detected by LC-MS (*n* = 6). Data are shown as the mean ± SEM. * p < .05, ** p < .01, *** p < .001 vs. the indicated groups. (k) The schema outlined the role of gut microbiota in TMAO generation induced by PSRC1 knockout.

Subsequently, we analyzed fecal and plasma microbial metabolites using untargeted metabonomics. Latent structure discriminant analysis (PLS-DA) revealed a remarkable difference in the metabolic profiles of feces and plasma samples (Figure 5a). A total of 109 fecal metabolites and 50 plasma metabolites were differentially abundant (p < .05) between the two groups (Figure S6a). Functional analysis suggested that 8 metabolic pathways were congruently enriched in plasma and fecal samples (Figure S6b). PSRC1 deletion caused a 30% reduction of lipid excretion in the fecal metabolites (Figure 5b), as well as the levels of bile acids (including allocholic acid and taurochenodeoxycholic acid), 25-hydroxycholesterol, acetoacetic acid and N-acetyl-a-neuraminic acid (NANA) (Figure 5c).

Remarkably, PSRC1 deletion increased the plasma levels of TMA-containing compounds, especially betaine (Figure 5d), and betaine was identified as the most significant taxonomic marker that distinguished PSRC1-knockout mice from apoE^−/−^ controls in the present results (Figure 5e). The analyses of the relationship between plasma and fecal metabolites generated by the gut microbiota revealed that plasma betaine levels were highly correlated with those in concurrently collected fecal betaine (Figure 5f). Consistently, both plasma TMAO and TMA levels were increased in DKO mice (Figure 5g). Additional analyses of individual taxa proportions revealed positive correlations with the plasma levels of atherogenic metabolites. For example, the plasma levels of TMAO, TMA and betaine were positively correlated with the abundances of TMA-producing bacteria as well as the enzymes involved in TMA synthesis, including betaine reductase, betaine-aldehyde dehydrogenase, cholinesterase activity and *PLD* (Figure 5h), suggesting the predominant roles of microbial enzymes in TMAO and betaine production in the murine PSRC1-knockout model (Figure 5i). In addition, we found that altered proportion of gut microbiota could contribute to the production of TMAO. The relative abundance of genera *Helicobacter, Acutalibacter, Lachnoclostridium* and *Eubacterium* was positively correlated with circulating TMAO levels, whereas *Turicimonas* and *Erysipelatoclostridium* exhibited the negative correlations (Figure S7).

To further verify the inhibitory effect of PSRC1 on choline consumption, both groups of mice were given a 1% choline diet for 8 weeks. We found that PSRC1 deletion significantly increased plasma TMA and TMAO levels by 1.51- and 2.26-fold, respectively, and these changes were abolished by antibiotic treatment (Figure 5j). These data revealed that the increased TMAO levels caused by PSRC1 deletion were gut microbiota dependent (Figure 5k).

### The atherosclerotic susceptibility due to PSRC1 deficiency is transferable by fecal transplantation

**Figure 6.**
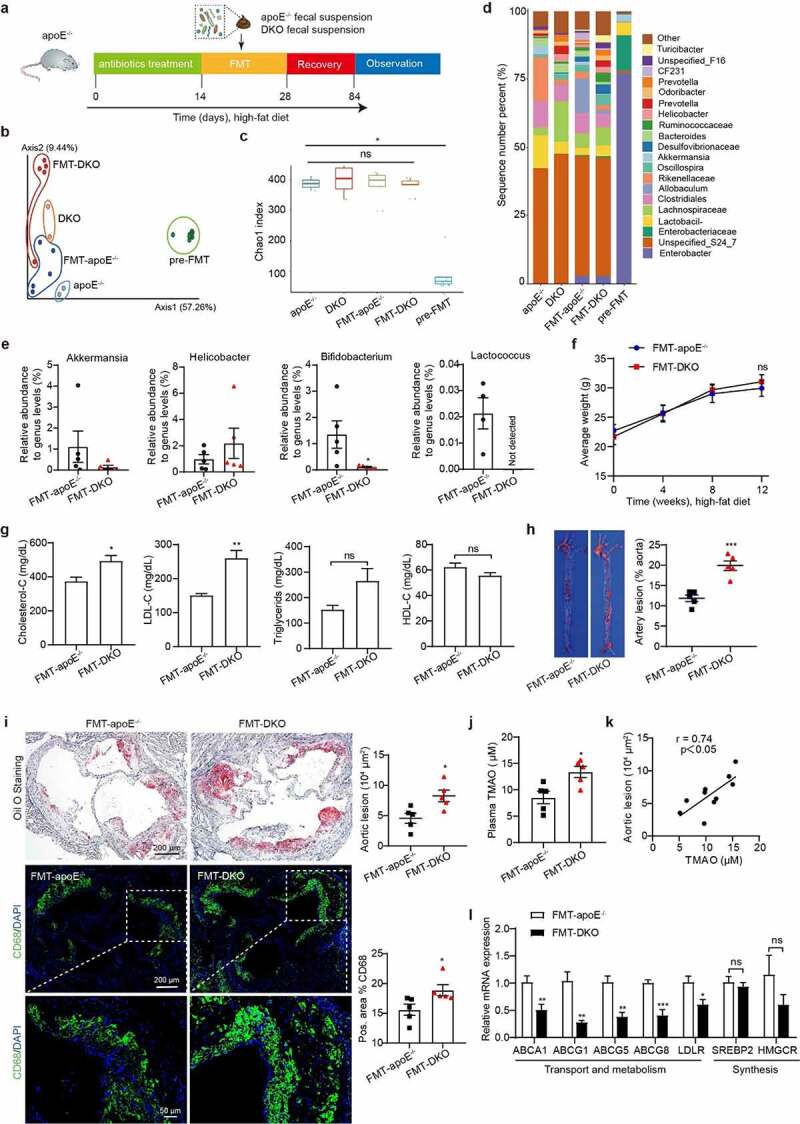
**Fecal transplants from PSRC1 knockout mice accelerated atherosclerotic plaque formation in HFD-fed apoE^−/−^ recipients**. (a) FMT experimental design protocol. 8-week-old male apoE^−/−^ mice were treated with antibiotics for 2 weeks and gavaged with the fecal suspension of either apoE^−/−^ or DKO donor mice for another 2 weeks. Both FMT-apoE^−/−^ (*n* = 5) and FMT-DKO (*n* = 5) were supplemented with HFD until 20 weeks of age. The V3-V4 region of the bacterial 16S rRNA gene was sequenced in fecal samples and the aortic plaque formation was evaluated. (b) MDS plot of a Bray-Curtis assessment of beta diversity. Box plots of the ANOSIM distances to pre-FMT and remaining four groups showed obvious differences in the gut microbiota composition. (c) Chao1 index for measuring alpha diversity among the pooled apoE^−/−^ (*n* = 2) and DKO (*n* = 2) samples, the apoE^−/−^ mice pre FMT (*n* = 10), the apoE^−/−^ mice after FMT with apoE^−/−^ (*n* = 5) and DKO (*n* = 5) feces. Significance of differences among the groups was assessed by Wilcoxon rank sum test. (d) Barplot of five groups showed the significant difference at the genus level. (e) Relative abundances of genera were significantly altered by PSRC1 deletion in the gut microbiota. (f) Body weight was measured every 4 weeks during the period of HFD (*n* = 5). (g) Comparison of plasma lipid profiles were determined (*n* = 5). (h) Representative images of oil red O-stained *en face* aortas from apoE^−/−^ that received feces from apoE^−/−^ or DKO mice after a 12-week HFD (*n* = 5). (i) The representative microphotographs of lipid area and CD68-positive macrophages in the aortic root sections by staining with oil red O and immunofluorescence (*n* = 5). (j) Plasma levels of TMAO were determined by LC-MS (*n* = 5). (k) Correlation between TMAO and aortic lesion was analyzed. Pearson correlation coefficients (r) and p values are presented in the graph. (l) Hepatic mRNA was assessed for cholesterol transport, metabolism and synthesis genes normalized to β-actin (*n* = 5). Data are shown as the mean ± SEM. * p < .05, ** p < .01, *** p < .001 vs. FMT-apoE^−/−^ mice.

We performed FMT to confirm the cause–effect relationship between PSRC1 deletion-induced dysbacteriosis and atherogenesis. Antibiotic-treated apoE^−/−^ mice received fecal microbiomes from 2 of the apoE^−/−^ and 2 of the DKO mice and were placed on a HFD for 12 weeks (Figure 6a). Bacterial 16S rRNA gene sequencing was used to assess the post-FMT microbiota profiles. Bray–Curtis analysis showed clear separation, and the post-FMT samples tended to cluster with those of the corresponding donors (Figure 6b). Following gastric gavage with a cocktail of antibiotics, alpha diversity in the pre-FMT group was markedly reduced, suggesting that pre-FMT antibiotic treatment of mice was successful; however, there were no significant differences between the two post-FMT groups (Figure 6c). At the genus levels, consistent modifications were found in the abundance of *Akkermansia* and *Helicobacter*; nevertheless, no significant differences were observed in the abundance of either of the above genera (Figures 6d and 6e). This unmarkable change was not surprising because the apoE^−/−^ and DKO mice in the initial study were not subjected to any intervention. The probiotics *Bifidobacterium* and *Lactococcus* were significantly depleted in the feces of the post-FMT-DKO mice compared with that of post-FMT-apoE^−/−^ mice (Figure 6e).

To clarify the role of dysbiosis induced by PSRC1 deletion in atherogenesis, plasma lipid levels and aortic plaques were evaluated in both groups treated with the atherogenic diet. We found that DKO recipients showed an elevation in total cholesterol (TC) and low-density lipoprotein-cholesterol (LDL-C) levels. However, no significant difference was found in body weight and plasma triglyceride (TG) and high-density lipoprotein-cholesterol (HDL-C) levels (Figures 6f and 6g). Plaque quantification revealed that DKO feces recipients showed larger plaque areas in *en face* aortas than apoE^−/−^ feces recipients (Figure 6h). We also found that DKO feces recipients displayed significantly increased lipid accumulation and macrophage infiltration in atherosclerotic plaques (Figure 6i). Notably, the circulating TMAO levels in DKO feces recipients were elevated and exhibited a positive correlation with aortic plaque areas (Figures 6j and 6k), indicating that the potential for TMAO generation resulting from PSRC1 deficiency could be transferred by fecal transplantation. To investigate the contributing mechanism by which PSRC1 deletion elevated plasma cholesterol levels, we determined the expression of cholesterol transport and synthesis genes in the liver. Feces from DKO mice caused a significant reduction in hepatic ABCA1, ABCG1, ABCG5 and ABCG8 as well as LDL receptor (LDLR) mRNA levels without affecting the expression of cholesterol synthesis genes (Figure 6l). Collectively, these results suggested that the regulation of the gut microbiota may be the main cause of the atherosclerosis-protective role of PSRC1 in this study.

## Discussion

Although previous studies have revealed a close relationship between PSRC1 polymorphisms and CAD,^11–13^ but the present findings revealed a completely new aspect of PSRC1 function. Using the apoE^−/−^ model of atherogenesis, we demonstrated that PSRC1 deletion increased systemic TMAO levels via i) enriched TMA-producing bacteria, ii) enhanced betaine and TMA synthesis, and iii) upregulated hepatic FMO3 expression. Transplantation of the gut microbiota from PSRC1-knockout mice elevated plasma TMAO levels and significantly accelerated HFD-induced atherogenesis. This study has broadened our understanding, indicating that the “PSRC1-gut microbiota-TMAO axis” is an alternative target for the atheroprotective effect of PSRC1. To the best of our knowledge, our current data are the first to define novel and direct effects of the host gene PSRC1 on the intestinal immune homeostasis, gut microbiome, hepatic FMO3 levels, and thus TMAO production and microbial dysbiosis-induced atherosclerosis.

In agreement with our previous study on the role of PSRC1 in atherosclerosis,^17^ double-knockout of PSRC1 and apoE significantly accelerated plaque formation compared with apoE knockout mice, reconfirming the atherosclerosis-protective effect of PSRC1. The successful mouse model is also a prerequisite for subsequent analysis. Our study demonstrated that the gut microbiota was an early and directly damaged “organ” after PSRC1 knockout, partly evidenced by the significantly disordered gut microbiome. We observed that three kinds of TMA-producing strains, *Clostridium-asparagiforme, Desulfovibrio-desulfuricans* and *Desulfovibrio-alaskensi*, were enriched in DKO mice, and these species have been reported to be classical bacteria governing choline-to-TMA transformation.^27,28^ To achieve detailed mechanistic insight into how gut microorganisms enriched by PSRC1 deletion contribute to the enrichment of specific metabolites, we attempted to understand the function of the gut microbiome. The microbial TMA lyases *Cut* and *Cnt* family genes encode the subunits of the oxidoreductase enzyme that is responsible for the conversion of choline and L-carnitine into TMA.^29^ However, microbiome sequencing showed a very low number of reads of the *Cut* and *Cnt* genes (on average of 20 and 3 RPKM, respectively) and no significant difference, which may be attributed to the normal chow diet used in the present results but not the choline-supplemented diet. Recent study reported that a novel microorganism enzyme, *PLD*, exists in non-choline-metabolizing bacteria and could liberate choline from phosphatidylcholine.^30^ The microbiome in PSRC1-knockout mice showed a higher abundance of *PLD* than that in controls. Based on this finding, it is possible that due to the conversion mediated by the *PLD*-encoded enzyme, the content of choline (a TMA precursor) is much more than the original 0.2%. However, the species and distribution of this enzyme in commensal gut strains have not yet been identified in mice. A previous study also reported that the TMA precursors could be converted into TMA via various enzymes.^31^ From the abundance of genes encoding TMA synthesis, we inferred that dietary choline was converted into betaine in the present study via the sequential action of choline dehydrogenase and betaine-aldehyde dehydrogenase activity, and betaine was transformed into TMA by the action of betaine reductase (Figure 5i). In addition, the increased activity of cholinesterase and acetylglycerophosphocholine esterase may have been involved in the transformation between choline and phosphatidylcholine. The results unveil a role for PSRC1 deletion in the conversion of choline to TMA independent of the *CutC*-encoded TMA lyase.

The generation of TMAO is modifiable by dietary consumption, the gut microbiota and hepatic FMO3 activity. Surprisingly, it is observed that PSRC1 deletion increased FMO3 expression. FMO3, which has 10-fold higher specific activity in the liver than FMO1, is the main enzyme responsible for the conversion of TMA into TMAO.^24^ Previous study has confirmed the binding of ERα in the promoter region of FMO3 in mice as well as in the first intron of FMO3 in human.^25^ RNA-seq revealed a downregulation of FMO4 and an upregulation of ERα in estrogen-treated chicken livers.^32^ Notably, our sequencing data also showed a reduction in ERα levels following PSRC1 knockout and exhibited a negative correlation with FMO3 expression in both male and female mice. Congruent to these findings, our work further demonstrated a causality between upregulated FMO3 and ERα using an ERα-specific inhibitor. Detailed mechanism by which PSRC1 deficiency regulates ERα-FMO3 signaling warrants further study. For example, the direct binding of ERα in the promoter region of FMO3,^25^ the degradation of ERα protein,^33^ or the increased expression of ERα-targeted miRNA should be considered.^34^

Another significant piece of evidence regarding the unfavorable effect of PSRC1 deletion is the increased plasma TMAO and betaine production. TMAO is a gut microbiota-dependent metabolite generated from choline, phosphatidylcholine, carnitine and betaine. Elevated plasma levels of choline, TMAO and betaine have been demonstrated to be positively associated with MACEs.^8^ Our study revealed that PSRC1 deficiency significantly increased the plasma betaine levels, which was the most significant taxonomic marker that distinguishes the two groups. We further performed correlation analyses between metabolomics and metagenomics and identified TMA-producing bacteria, including *Desulfovibrio-desulfuricans* and *Clostridium-asparagiforme*, and TMA-related enzymes, including betaine-aldehyde dehydrogenase, betaine reductase, cholinesterase and *PLD*, as being highly correlated with the generation of TMAO and betaine. This finding suggests that PSRC1 deletion has a direct impact on microbial TMAO production in the murine gut.

Consistently, PSRC1 deficiency markedly increased plasma TMA and TMAO levels in choline-fed mice. More importantly, this promotion of TMAO production was moderately elevated than that in chow diet-fed mice (1.42-fold increase in choline-supplemented mice and 1.25-fold in chow-diet mice), implying that the inhibitory capacity of PSRC1 on TMAO may increase with an overdose of choline. We also found that the antibiotic treatment could decrease TMAO levels increased by PSRC1 deletion by inhibiting the growth of most gut microorganisms. Indeed, long-term use of antibiotics may cause severe microbial gut dysbiosis and is not suitable for alleviating atherosclerosis.^35^ Overall, these results reconfirmed that the gut microbiota is a potential target in PSRC1 knockout-induced atherosclerosis through its impact on TMAO generation.

In addition, the effect of PSRC1 deletion on other bacterial species and metabolites likewise indicated that PSRC1 is a protective factor. For example, *A. muciniphila, Mucispirillum* and *Adlercreutzia* were markedly depleted in the gut of DKO mice. *A. muciniphila*, as a next-generation beneficial microbe, has reduced plasma cholesterol and inflammation levels, improved insulin sensitivity and ameliorated atherosclerotic lesion areas in animal and human studies.^36^ *Adlercreutzia* inhibits hyperlipidemia and obesity,^37^ while *Mucispirillum* plays a role in inhibiting inflammation.^38^ Conversely, the enriched genus *Helicobacter* may become an opportunistic pathogen, which would cause pro-inflammatory cytokine production and hyperlipidemia.^39^ PSRC1 deletion also activates urease and its accessory protein *UreE* and enriches the proton-gated inner membrane channel protein *UreI*, all of which favor *H. pylori* survival in acidic environments. ArgG, a gene encoding argininosuccinate synthase, is a crucial rate-limiting enzyme involved in arginine synthesis and the uric acid cycle.^40^ The sequencing data showed that PSRC1 deletion downregulated ArgG levels, which may reduce the tolerance of beneficial bacteria to acid stress and thus inhibit growth performance.

It has been reported that apoE^−/−^ mice exhibited an elevation in TMAO levels and atherosclerotic plaque areas when they received cecal microbes from high TMAO-producing strain mice compared with that from low TMAO-producing strain mice,^5^ indicating that the susceptibility of TMAO generation and atherosclerosis could be transferable via FMT. Consistent with previous studies, gavage with PSRC1-knockout mouse feces resulted in higher plasma TMAO levels in apoE^−/−^ mice. This observation was also accompanied by a reduction in the abundance of beneficial commensal bacteria, such as *Bifidobacterium* and *Lactococcus*. Parallel studies showed that the transfer of the microbiota from PSRC1-knockout mice to antibiotic-treated animals resulted in significantly increased lipid levels and macrophages infiltration compared to that with an equivalent transfer from apoE^−/−^ mice, which implying that the regulation of PSRC1 on the gut microbiota might predate the effects of PSRC1 on lipid metabolism and inflammation. A positive correlation between plasma TMAO levels and accelerated atherosclerotic lesion in recipients clearly demonstrated that the beneficial role of PSRC1 was, at least in part, attributable to the microbiome. However, further studies are required to investigate whether this aggravation resulted from a single genus/species, a consortium in the gut microbiota, or infectious agents beyond the impact of TMAO.

The identity of the host receptors or sensors that contribute to gut dysbiosis is remarkably complex and unclear. Our previous studies indicated that intravenous injection of a PSRC1-overexpressing construct inhibited NF-κB-mediated inflammation.^17^ PSRC1 also acts as a regulator of plasma progranulin.^18^ As we reviewed elsewhere, progranulin is a secreted protein with important functions in immune and inflammatory processes.^41^ NLRP3 inflammasome-knockout mice exhibited significant differences in their gut microbiota composition compared with wild-type (WT) mice,^20^ indicating an important role of the immune-inflammatory system in the regulation of intestinal barrier function and gut microbiota composition. Therefore, one can speculate that colonic PSRC1 deletion may disturb the gut microbiota by altering the colon immune-inflammatory response, as evidenced by the elevated proinflammatory phenotypes and downregulated anti-inflammatory markers. The significant correlations between the colonic cytokines and gut microbiota constituents highlight the potential causality. Further studies are needed to purify and characterize colonic macrophages or T lymphocytes from colonic tissues in murine model.

This study demonstrates that deficiency of PSRC1 can directly target gut microbiota and hepatic FMO3, thus accelerating TMAO production and atherogenesis. However, some limitations remain to be investigated. Firstly, we performed the study using double-knockout mouse model to describe the metagenome signature of PSRC1 deletion in the context of atherosclerosis; however, the response may not exclude the pro-inflammatory environment induced by apoE deficiency. Secondly, further study is warranted to confirm our data in a clinical setting since the murine model does not completely mirror the gut microbiota condition in patients with atherosclerosis. Moreover, the exact mechanism by which the PSRC1 knockout–colonic inflammation axis regulates the gut microbiota still required further study. Whether circulating Ly6C^high^ monocytes-mediated immunomodulation contributes to macrophages infiltration and inflammatory response in colon and atherosclerotic plaque remains to be investigated. In summary, our work sheds light on the influence of PSRC1 on atherosclerosis in the mouse models independent of the known lipid metabolism and inflammatory pathways. This study has broadened our horizons that microbial gut dysbiosis, as the upstream regulator of lipid metabolism and inflammatory response, may become a new risk factor and therapeutic target; while PSRC1, as a crucial regulator of gut microbiota, plays an indispensable role in maintaining intestinal homeostasis, reducing TMAO levels and alleviating atherogenesis.

## Materials and methods

### Animal model

Animal protocols were approved by the Animal Experiment Committee of Nanfang Hospital at Southern Medical University (Guangzhou, China). All diets, including the normal chow diet (containing 0.2% total choline, 33.58% corn, 25% flour, 17.5% soybean, 13.3% wheat flour, 4.2% fish meal, 2.5% soybean oil, 2% dicalcium phosphate, 1.3% calcium carbonate, 0.3% salt, 0.08% minerals, 0.04% vitamin), choline-supplemented diet (1% choline) and HFD (15% fat and 1.2% cholesterol), were acquired from Guangdong Medical Laboratory Animal Center (Guangdong, China). ApoE^−/−^ mice on a C57BL/6 J background were purchased from Viewsolid Biotech Co., Ltd. (Beijing, China). PSRC1^−/−^ mice were generated at Viewsolid Biotech Co., Ltd. using the CRISPR–Cas9 system to delete exon 4. PSRC1^−/−^ C57BL/6 J background mice were bred onto the apoE^−/−^ strain for at least 5 generations. The PSRC1^−/−^apoE^−/−^ offspring, termed DKO mice in this study, were genotyped by PCR amplification of tail DNA (Figure S8). The genotyping primers were used to amplify a 302-bp product corresponding to the PSRC1 mutant allele and a 1060-bp fragment corresponding to the WT allele, while a 346-bp product corresponding to the apoE mutant allele and a 428-bp fragment corresponding to the WT allele. PCR conditions consisted of an initial denaturation at 94°C for 2 min, followed by 35 cycles of 98°C for 10s, 60°C for 30s, and 68°C for 60s. The guide RNA (gRNA) sequences used for the CRISPR protocol and genotyping primer sequences are presented in Supplementary Material Table3.

### DNA extraction and metagenomic sequencing

Murine fecal samples were freshly collected from 8-week-old chow diet-fed apoE^−/−^ and DKO mice and frozen immediately, and genomic DNA was extracted. Paired-end metagenomic sequencing was performed on an Illumina NovaSeq with an insert size of 350 bp for each sample, followed by high-throughput sequencing with reads with a length of 2 × 150 bp. The sequencing reads were quality controlled (Trimmomatic; ILLUMINACLIP: adapters_path: 2: 30: 10; SLIDINGWINDOW: 4: 20; MINLEN: 50), and high-quality reads were obtained by filtering low-quality reads with ambiguous “N” bases, adaptor contamination, and DNA contamination from the Illumina raw reads; and by trimming low-quality terminal bases of reads simultaneously. Over 80% of the clean reads remained (approximately 18.2 million reads per sample). To better present the difference between apoE^−/−^ and DKO mice, PCoA was performed at the genus level based on Bray–Curtis distance. Gene functional annotations were assessed by BLASTP search (e-value < 10^–5^) with the eggNOG and KEGG databases.

### Untargeted metabolomics

Fecal samples (100 mg) were vortexed, ground and ultrasonicated in succession after adding 0.6 mL of 2-2-chlorophenylalanine (4 ppm) in methanol, while 100 μL plasma samples were vortexed after adding 400 µL of methanol. Following centrifugation at 4°C for 10 min at 12 000 rpm, all supernatant was filtered through a 0.22 μm membrane. Then, 20 µL of filtrate from each sample was used for the quality control (QC) samples, and the remaining samples were used for stable isotope dilution high-performance liquid chromatography-tandem mass spectrometry (LC–MS) detection. Chromatographic separation was accomplished in a Thermo Ultimate 3000 system equipped with an ACQUITY UPLC® HSS T3 (150 × 2.1 mm, 1.8 µm, Waters), and the ESI-MSn experiments were executed on a Thermo Q Exactive Plus mass spectrometer with spray voltages of 3.5 kV and −2.5 kV in positive and negative modes, respectively. The raw data were first processed with peak identification, peak filtration and peak alignment, and the mass-to-charge ratio (m/z), retention time and intensity values were obtained. The identification of metabolites was first confirmed in terms of precise molecular weight (error < 30 ppm) and then annotated using the five most relevant databases: Human Metabolome Database (HMDB) (http://www.hmdb.ca), METLIN (http://metlin.scripps.edu), Massbank (http://www.massbank.jp/), LipidMaps (http://www.lipidmaps.org) and mzClound (https://www.mzcloud.org). Principal component analysis (PCA) and projection to PLS-DA score plots were performed to show the separation of the groups.

### Antibiotic treatment and fecal microbiota transplantation studies

Fecal samples were collected from 8-week-old apoE^−/−^ and DKO mice, placed into tubes containing freezing solution (sterile PBS with 12.5% glycerol) and homogenized. The suspended pellets were stored at −80°C until utilized. The antibiotic protocol was conducted as previously described.^20^ Briefly, 8-week-old apoE^−/−^ mice were gavaged daily with antibiotics (ampicillin 1 g/L, metronidazole 1 g/L, vancomycin 1 g/L and neomycin 1 g/L) on days 1–14. Then, the mice were randomized into two groups (n = 5 per group), namely, the FMT-apoE^−/−^ and FMT-DKO groups, and FMT was performed on days 15–28 via oral gavage with the fecal suspension. The gut microbial composition determined using 16S ribosomal RNA gene sequencing and atherosclerotic plaques were compared after a continued HFD for 12 weeks.

### 16S ribosomal RNA gene sequencing analysis

The V3-V4 region of the bacterial 16S rRNA gene was detected by PCR (98°C for 1 min, followed by 30 cycles at 98°C for 10s, 50°C for 30s, and 72°C for 30s, and a final extension at 72°C for 5 min) using the primers: 338 F, 5’- ACTCCTACGGGAGGCAGCAG −3’, and 806 R, 5’- GGACTACHVGGGTWTCTAAT −3’. Raw FASTQ files were demultiplexed and quality filtered using QIIME (version 1.17). Operational taxonomic units (OTUs) were clustered with a 97% similarity cutoff using UPARSE, and chimeric sequences were identified and removed using UCHIME. The phylogenetic affiliation of each 16S rRNA gene sequence was analyzed by RDP Classifier against the SILVA (SSU123) 16S rRNA database using a confidence threshold of 70%. To examine dissimilarities in community composition, we performed PCA in QIIME.

### RNA-seq

Total RNA was extracted using RNAex Pro Reagent (Accurate Biotech, Hunan, China). The RNA concentrations and integrity parameters were as follows: A260/A280 ratio > 1.8, A260/A230 ratio > 2.0 and RIN value > 7.0. Library construction and sequencing were performed by GuoKe Biotec (Beijing, China). Sequencing was conducted on the Illumina NovaSeq 6000 system and 42.67 Gb of clean data were acquired. The criteria for differentially expressed genes were fold change > 2 and adjusted p value < .05.

### Cell culture, treatments, and transfection

The alpha mouse liver 12 (AML12) cell line was cultured in DMEM/F12 (1:1) supplemented with 10% fetal bovine serum (FBS), 1% ITS liquid medium supplement and 40 ng/mL dexamethasone. The medium was changed to serum-free conditions for 8 h before any treatment.

The target sequence of the mouse PSRC1 gene was obtained from Genbank, and the comparison of positive sequence clones was as follows: ATGGAGGATCTGAAAGAGGATATCAAGTTCATTGTG

GACGAGACCTTGGACTTCGGAGGGCTGTCTCCATCTGACAGTCATGAGGAAGAAGACATAACAGTATTAGTGAGTCCAGAGAAACCACTTCGACGGGGCCTCGCCCATCGGAGTAACCCAAATGAAGTAGCTCCCGCCCTCCAGGGTGTGCGGTTTAGCTTGGGCCCGCTCAGCCCAGAGAAGCTGGAAGAGATTCTTGATGAAGCCAACCGCCTGGCGGCTCAGCTGGAGGAGTGTGCCCTGAAAGATCGGGAGAGGGCTGGTACAGGCCCTGGAAGGCCCAGCCCCAGAGGGAAACCCAGTCCTCGGCGGGAGACCTTCGTCCTGAAGGATAGCCCTGTCCGAGATCTGCTGCCCACCGTGAGTTCTTGGAGCACCCCACCTCCAAGCAGCCTAGCTGGGCTCCGGAGCAGTGATAAAAAGGGGTCAGCCAGGGCTGTCCGGGTGGCATCCGGAAAGAAGCCCTCCAGCATAAAGAAGGAATCACCCACTTGCAATCTGTTCCCTGCATCCAAAAGCCCGGGGCGCTCTCCTCTTGCACAACCAATTCTTCCACCTCGGCGGAAAACTGGGTTCGGTGCCCGGACAACAGCAAGCCCACCAATTCCTGTCAGACCAGTTCCACAGTCCTCAGCTAGCAACTCCCAATGTTCATCCCGGCTCCAGGGAGCAGCTGTCAAGTCTTCCAGTCGACTCCCTGTCCCTTCAGCCATCCCCAAGCCTGCCACCCGAGTGCCACTCATTGGGCGGAGTCTACCACCTGGAAAAGGTGCCCTAGCTCCAGATTCTCTCTCAACTCAGAAAGGGCATCCAAGCGCCATAGGGCACAGAGCCTCTGTTTCCCAGAAAACAAACCTTCCAACCACCAGTGCGGCTCGAGGCAGGACCACCAGTGCCGCTCGAGGCAGGGCGCAGCCCCTCAGGAAAGCTGCAGTCCCTGGACCGACTAGG.

The plasmid was subcloned into the adenovirus shuttle plasmid vector (CMV-MCS-3FLAG-SV40-EGFP). Cells were transfected with a recombinant adenovirus encoding PSRC1 (PSRC1 OE) or a negative control (NC) for 48 h – 72 h. The transfection efficiency was analyzed by quantitative real-time polymerase chain reaction (qRT–PCR) and Western blotting.

### Plasma analysis

The levels of TMAO and TMA in plasma samples were determined by LC–MS using a trimethylamine-d9 *N*-oxide TMAO (d9-TMAO)-labeled internal standard as previously described.^42^ The levels of TG, TC, LDL-C and HDL-C were measured using assay kits (Nanjing Jiancheng Bioengineering Institute, China).

### Atherosclerotic lesion analysis

For *en face* staining of the plaque areas in mice, longitudinally opened aortas were fixed with 4% paraformaldehyde for 24 h and then stained with oil red O. To detect the lipid areas of aortic roots, the upper half of the heart, including the aortic root, was embedded in optimal cutting temperature (OCT) compound and then cryosectioned for further oil red O staining. In addition, aortic roots were embedded in paraffin and then sectioned for further staining with hematoxylin and eosin (HE) (anuclear, afibrotic and eosin-negative areas indicate the necrotic core areas), Masson’s trichrome (aniline blue indicates the collagen areas) and immunofluorescence (IF). IF was performed with rat anti-F4/80 (1:200, ab6640), rabbit anti-iNOS (1:400, ab178945), rabbit anti-pNF-κB p65 (1:1000, 3033S) and rabbit anti-CD68 (1:200, 97778S) antibodies. Primary rabbit antibodies were visualized with Alexa Fluor 488-conjugated secondary antibodies, rat antibodies were visualized with Cy3-conjugated antibodies (both from Beyotime, China), and fluorescence measurements were performed using a TCS SP8 confocal laser scanning microscope (LEICA, Germany).

### Quantitative real–time PCR

Total mRNA was extracted with TRIzol reagent from cell lysates or tissues according to the manufacturer’s instructions. cDNA was synthesized using a PrimeScript™ RT Reagent Kit (TaKaRa, China). qRT–PCR was performed on a LightCycler 480 II (Roche, Switzerland) using a SYBR Premix Ex Taq Kit (TaKaRa, China). The results were normalized based on the housekeeping gene β-actin. The quantitative measurements were determined using the 2^−ΔΔCT^ method. All primer sequences were designed using PrimerBank software and are listed in Supplementary Material Table 3.

### Protein isolation and Western blot analysis

Protein was extracted by homogenization in lysis buffer according to the product information manual. Then, the protein was separated using 8% or 10% SDS–PAGE gels and transferred to polyvinylidene fluoride membranes (Millipore, Burlington, MA). Next, the membranes were blocked in Tris-buffered saline/Tween (TBST) containing 5% nonfat dry milk for 1 h at room temperature and then incubated with the following primary antibodies for 14 h – 18 h at 4°C: anti-β-tubulin (66240–1; 1:50000) (Proteintech); anti-PSRC1 (GTX128047; 1:1000) (GeneTex); anti-FMO3 (ab126711; 1:4000) and anti-ERα (ab32063; 1:1000). Finally, the membranes were incubated with secondary HRP-linked anti-mouse IgG (FDM007; 1:10000) or HRP-linked anti-rabbit IgG (FDR007; 1:10000) (Fude Biotech, Hangzhou, China), and detection was performed using an ECL detection kit (Thermo Fisher Scientific, Rockford, USA). ImageJ software was used to measure the protein band intensity, and the expression of specific proteins was normalized based on β-tubulin.

### Statistical analyses

Unpaired t tests or Mann–Whitney *U* tests were used to compare two groups of continuous variables. One-way analysis of variance (ANOVA) was used for comparisons among multiple groups, and post hoc analysis was further used for two–group comparisons. Pearson correlation analysis was used to evaluate the association between two continuous variables. The results were analyzed using SPSS v13.0. All data were presented as mean ±SEM, and P < .05 (two-tailed) was considered statistically significant.

## Supplementary Material

Supplemental Material

## Data Availability

All data generated in this study are included in this article and supplementary information or are available from the corresponding author on reasonable request. The raw metagenomic sequences are deposited at NCBI’s BioProject database under accession PRJNA839170.
