# Supplementary material for: Deficiency of PSRC1 accelerates atherosclerosis by increasing TMAO production via manipulating gut microbiota and flavin monooxygenase 3
Source: Gut Microbes. 2022 May 25;14(1):2077602. doi: 10.1080/19490976.2022.2077602 (PMC9135421; doi:10.1080/19490976.2022.2077602)
Supplement: Supplemental Material [file KGMI_A_2077602_SM2619.zip › Supplementary Table 1.docx]

**Supplemental Table 1: Relative abundance (%) of specific Helicobacter species**

| Helicobacter species | SUM  Abundance | apoE^-/-^ mice | | | | | | DKO mice | | | | | |
| --- | --- | --- | --- | --- | --- | --- | --- | --- | --- | --- | --- | --- | --- |
|  |  | ① | ② | ③ | ④ | ⑤ | ⑥ | ① | ② | ③ | ④ | ⑤ | ⑥ |
| Helicobacter_typhlonius | 57944 | 0 | 0 | 0 | 0 | 0 | 0 | 0.14680 | 0.32786 | 0.80508 | 0.30839 | 0.38249 | 0.17707 |
| Helicobacter_rodentium | 52254 | 0 | 0 | 0 | 0 | 0 | 0 | 0.13642 | 0.28688 | 0.77546 | 0.27000 | 0.34218 | 0.16598 |
| Helicobacter_hepaticus | 35324 | 0 | 0 | 0 | 0 | 0 | 0 | 0.09816 | 0.20798 | 0.51263 | 0.17476 | 0.23064 | 0.11140 |
| Helicobacter_apodemus | 22207 | 0 | 0 | 0 | 0 | 0 | 0 | 0.06002 | 0.11532 | 0.32703 | 0.12411 | 0.14990 | 0.06591 |
| Helicobacter_pullorum | 2756 | 0 | 0 | 0 | 0 | 0 | 0 | 0.00774 | 0.01518 | 0.04004 | 0.01412 | 0.01791 | 0.00925 |
| Helicobacter_fennelliae | 2401 | 0 | 0 | 0 | 0 | 0 | 0 | 0.00636 | 0.01565 | 0.03861 | 0.00832 | 0.01451 | 0.00680 |
| Helicobacter_muridarum | 1371 | 0 | 0 | 0 | 0 | 0 | 0 | 0.00439 | 0.00613 | 0.01944 | 0.00863 | 0.00897 | 0.00462 |
| Helicobacter_canadensis | 1105 | 0 | 0 | 0 | 0 | 0 | 0 | 0.00242 | 0.00613 | 0.01797 | 0.00482 | 0.00655 | 0.00370 |
| Helicobacter_winghamensis | 889 | 0 | 0 | 0 | 0 | 0 | 0 | 0.00297 | 0.00437 | 0.01266 | 0.00495 | 0.00680 | 0.00204 |
| Helicobacter_pametensis | 760 | 0 | 0 | 0 | 0 | 0 | 0 | 0.00226 | 0.00525 | 0.01093 | 0.00358 | 0.00416 | 0.00251 |
| Helicobacter_jaachi | 739 | 0 | 0 | 0 | 0 | 0 | 0 | 0.00255 | 0.005 | 0.01026 | 0.00309 | 0.00401 | 0.00292 |
| Helicobacter_suis | 612 | 0 | 0 | 0 | 0 | 0 | 0 | 0.00192 | 0.00456 | 0.00884 | 0.00163 | 0.00372 | 0.00221 |
| Helicobacter_himalayensis | 516 | 0 | 0 | 0 | 0 | 0 | 0 | 0.00154 | 0.00226 | 0.00812 | 0.00301 | 0.00318 | 0.00149 |
| Helicobacter_cinaedi | 500 | 0 | 0 | 0 | 0 | 0 | 0 | 0.00146 | 0.00273 | 0.00636 | 0.00380 | 0.00289 | 0.00183 |
| Helicobacter_canis | 50 | 0 | 0 | 0 | 0 | 0 | 0 | 0 | 0.00040 | 0.00101 | 0.00053 | 0 | 0 |
| Helicobacter_pylori | 12 | 0 | 0 | 0 | 0 | 0 | 0 | 0 | 0.00045 | 0 | 0 | 0 | 0 |

Male apoE^-/-^ and DKO mice fed a chow diet, and at 8 weeks of age, feces were collected. Bacterial taxonomic levels were detected by metagenomics sequencing and 16 species within Helicobacter were only detected from the feces of DKO mice. Each taxon expressed as a percentage to the total bacterium population.
