# Supplementary material for: Deficiency of PSRC1 accelerates atherosclerosis by increasing TMAO production via manipulating gut microbiota and flavin monooxygenase 3
Source: Gut Microbes. 2022 May 25;14(1):2077602. doi: 10.1080/19490976.2022.2077602 (PMC9135421; doi:10.1080/19490976.2022.2077602)
Supplement: Supplemental Material [file KGMI_A_2077602_SM2619.zip › Supplementary Table 2.docx]

**Supplemental Table 2: Relative abundance (RPKM) of urease-specific genes of *H.pylori***

| **Gene** | apoE^-/-^ mice | | | | | | DKO mice | | | | | |
| --- | --- | --- | --- | --- | --- | --- | --- | --- | --- | --- | --- | --- |
|  | ① | ② | ③ | ④ | ⑤ | ⑥ | ① | ② | ③ | ④ | ⑤ | ⑥ |
| ureA | 7.87 | 54.11 | 5.34 | 34.49 | 8.45 | 64.56 | 5.09 | 16.34 | 16.44 | 9.47 | 16.98 | 13.02 |
| ureB | 0 | 31.45 | 0 | 22.30 | 0 | 32.89 | 20.03 | 11.83 | 18.33 | 23.68 | 36.10 | 29.71 |
| ureC | 35.74 | 104.47 | 39.86 | 72.74 | 77.14 | 175.11 | 72.80 | 56.10 | 66.27 | 54.31 | 62.51 | 66.21 |
| ureE | 2.31 | 0 | 0 | 0 | 1.34 | 0 | 5.21 | 4.64 | 4.61 | 0 | 6.12 | 4.95 |
| ureF | 0.41 | 1.26 | 1.24 | 0.53 | 0 | 1.23 | 4.88 | 1.99 | 0.47 | 0 | 5.44 | 2.65 |
| ureG | 88.59 | 173.19 | 94.17 | 139.75 | 130.37 | 283.92 | 107.08 | 68.93 | 81.35 | 69.33 | 70.92 | 90.45 |
| ureH | 0.62 | 0.30 | 0.29 | 0.43 | 0 | 0.88 | 14.43 | 1.98 | 2.44 | 1.02 | 6.97 | 1.69 |

Male DKO and apoE^-/-^ mice were fed a chow, and at 8 weeks of age, feces were collected. Bacterial taxonomic levels were detected by metagenomics sequencing and the genus *Helicobacter* was only detected from the feces of DKO mice. Although *H.pylori* was found in DKO②, the expression of urease genes and accessory proteins bloom in almost DKO mice. RPKM indicates reads per kilobase per million mapped reads; urea, ureB: urease subunit; ureC: pecific H. pylori nucleic acid sequence; ureE, ureF, ureG, and ureH: urease accessory proteins; ureI: pH-gated inner membrane channel.
