# Supplementary material for: Deficiency of PSRC1 accelerates atherosclerosis by increasing TMAO production via manipulating gut microbiota and flavin monooxygenase 3
Source: Gut Microbes. 2022 May 25;14(1):2077602. doi: 10.1080/19490976.2022.2077602 (PMC9135421; doi:10.1080/19490976.2022.2077602)
Supplement: Supplemental Material [file KGMI_A_2077602_SM2619.zip › Supplementary Table 3.docx]

**Supplemental Table 3: Primers**

| Primers used for genotyping | | |
| --- | --- | --- |
| Gene | Forward (5’-3’) | Reverse (5’-3’) |
| PSRC1^-/-^ | TTTGAGATCATTCTGTGCTAT | TCCTCACATGTAGGTCACAAG |
| apoE^-/-^ | TGCCTAGTCTCGGCTCTGAACTAC | CAACCTGGGCTACACACTAATTGAG |
| Primers used for qRT-PCR | | |
| Mouse Primers | | |
| Gene | Forward (5’-3’) | Reverse (5’-3’) |
| ACTIN | GTGCTATGTTGCTCTAGACTTCG | ATGCCACAGGATTCCATACC |
| PSRC1 | TCAAGTTCATTGTGGACGAGAC | GGGAGCTACTTCATTTGGGTT |
| NOS2 | ATCTTGGAGCGAGTTGTGGATTGTC | CTGGGAGGAGCTGATGGAGTAGTAG |
| Arg1 | ATGCTCACACTGACATCAACACTCC | GTCTCTTCCATCACCTTGCCAATCC |
| IL-1β | TGATGTGCTCACTGCCTGGTTTC | GTTGATGTGCTGCTGCGAGATTTG |
| IL-6 | CTCCCAACAGACCTGTCTATAC | CCATTGCACAACTCTTTTCTCA |
| IL-4 | TACCAGGAGCCATATCCACGGATG | TGTGGTGTTCTTCGTTGCTGTGAG |
| IL-13 | ACCCTTAAGGAGCTTATTGAGG | ATTGCAATTGGAGATGTTGGTC |
| TGF-β | CCAGATCCTGTCCAAACTAAGG | CTCTTTAGCATAGTAGTCCGCT |
| IL-10 | TTCTTTCAAACAAAGGACCAGC | GCAACCCAAGTAACCCTTAAAG |
| IL-17A | GAGCTTCATCTGTGTCTCTGAT | GCCAAGGGAGTTAAAGACTTTG |
| TNF-α | ATGTCTCAGCCTCTTCTCATTC | GCTTGTCACTCGAATTTTGAGA |
| Ym-1 | AAGACATTCCAAGGCTGCTACTCAC | GAAGGGTCACTCAGGATAAAGGTATGC |
| Ym-2 | TCTTGTCACAGGTCTGGCAATTCTTC | ATGTCTGACGGTTCTGAGGAGTAGAG |
| FMO3 | GGAACCAGGAATATGGAAG | GGTGACC TTCTGAGCTACAT |
| ERα | CTACTACCTGGAGAACGAGC | GCGTCGATTGTCAGAATTAGAC |
| BSEP | GTGTCTACTTCATGCTTGTGAC | GAGACTTAGATCGTTGACGGAT |
| FXR | GCAACCAGTCATGTACAGATTC | TTATTGAAAATCTCCGCCGAAC |
| ABCA1 | AGAAGGAGGCTCGGCTGAAGG | GAGGGATGAGGCTGCTAACAAACC |
| ABCG1 | CATGCTGCTGCCTCACCTCAC | TCTCGTCTGCCTTCATCCTTCTCC |
| ABCG5 | CATTGAAAGAGCACGATACCTG | AGATTCTGAACGAGACGCATAA |
| ABCG8 | GGACAAATTTGGATAAATGGGC | GATTACGTCTTCCACCCGTTT |
| SREBP2 | CCGCCTCCTTCTCCTTCTCCTC | CCGCAGTTTCACCATAGCCAGAG |
| LDLR | GAGGGACACGAGCAGGAGAGAG | CAGCAAAGCGGAAACACAGGAAAC |
| HMGCR | TGGCAGGACGCAACCTCTAT | TGACGGCTTCACAAACCACA |
